# Unraveling genomic variation from next generation sequencing data

**DOI:** 10.1186/1756-0381-6-13

**Published:** 2013-07-25

**Authors:** Georgios A Pavlopoulos, Anastasis Oulas, Ernesto Iacucci, Alejandro Sifrim, Yves Moreau, Reinhard Schneider, Jan Aerts, Ioannis Iliopoulos

**Affiliations:** 1 Division of Basic Sciences, University of Crete Medical School, Heraklion 71110, Greece; 2 Institute of Marine Biology, Biotechnology and Aquaculture IMBBC-HCMR, Heraklion, Crete, Greece; 3 Laboratory of Environmental Toxicology and Aquatic Ecology, Ghent University, Ghent, Belgium; 4 ESAT-SCD/iMinds-KU Leuven Future Health Department, University of Leuven, Kasteelpark Arenberg 10, box 2446, 3001 Leuven, Belgium; 5 Luxembourg Centre for Systems Biomedicine (LCSB), University of Luxembourg, 7 avenue des Hauts-Fourneaux, L-4362 Esch sur Alzette, Luxembourg

**Keywords:** SNPs, SNVs, CNV, Structural variation, Sequencing, Genome browser, Visualization, Polymorphisms, Genome wide association studies

## Abstract

Elucidating the content of a DNA sequence is critical to deeper understand and decode the genetic information for any biological system. As next generation sequencing (NGS) techniques have become cheaper and more advanced in throughput over time, great innovations and breakthrough conclusions have been generated in various biological areas. Few of these areas, which get shaped by the new technological advances, involve evolution of species, microbial mapping, population genetics, genome-wide association studies (GWAs), comparative genomics, variant analysis, gene expression, gene regulation, epigenetics and personalized medicine. While NGS techniques stand as key players in modern biological research, the analysis and the interpretation of the vast amount of data that gets produced is a not an easy or a trivial task and still remains a great challenge in the field of bioinformatics. Therefore, efficient tools to cope with information overload, tackle the high complexity and provide meaningful visualizations to make the knowledge extraction easier are essential. In this article, we briefly refer to the sequencing methodologies and the available equipment to serve these analyses and we describe the data formats of the files which get produced by them. We conclude with a thorough review of tools developed to efficiently store, analyze and visualize such data with emphasis in structural variation analysis and comparative genomics. We finally comment on their functionality, strengths and weaknesses and we discuss how future applications could further develop in this field.

## Introduction

High throughput sequencing (NGS) techniques have brought a remarkable revolution in the field of biology and other closely related fields and have shaped a new trend of how modern biological research can be done at a large scale level. With the advances of these techniques, it is feasible nowadays to scan and sequence a whole genome or exome at a base pair level at a low error rate, in an acceptable time frame and at a lower cost.

Based on the first Sanger sequencing technique, the *Human Genome Project* (1990–2003), allowed the release of the first human reference genome by determining the sequence of ~3 billion base pairs and identifying the approximately ~25,000 human genes [1-3]. That stood as a great breakthrough in the field of comparative genomics and genetics as one could in theory directly compare any healthy or non-healthy sample against a golden standard reference and detect genetic polymorphisms or variants that occur in a genome. Few years later, as sequencing techniques became more advanced, more accurate and less expensive, the *1000 Human Genome Project*[4] was launched (January 2008). The main scope of this consortium is to sequence, ~1000 anonymous participants of different nationalities and concurrently compare these sequences to each other in order to better understand human genetic variation. Recently, as a result of the project, 1092 such human genomes were sequenced and published [5]. The *International HapMap Project* (short for “haplotype map”) [6-10] aims to identify common genetic variations among people and is currently making use of data from six different countries.

Shortly after the *1000 Human Genome Project*, the *1000 Plant Genome Project* (http://www.onekp.com) was launched, aiming to sequence and define the transcriptome of ~1000 plant species from different populations around the world. Notably, out of the 370,000 green plants that are known today, only ~125,000 species have recorded gene entries in GenBank and many others still remain unclassified [11]. While the *1000 Plant Genome Project* was focused on comparing different plant species around the world, within the *1001 Genomes Project*[12], 1000 whole genomes of *A. Thaliana* plants across different places of the planet were sequenced.

Similar to other consortiums, the *10,000 Genome Project*[13] aims to create a collection of tissue and DNA specimens for 10,000 vertebrate species specifically designated for whole-genome sequencing. In addition, the overarching goal of the *1000 Fungal Genome Project* (F1000 - http://1000.fungalgenomes.org) is to explore all areas of fungal biology by providing broad, genomic coverage of Kingdom Fungi. Notably, sequencing advances have paved the way to metagenome sequencing, which is defined as an approach for the study of microbial populations in a sample representing a community by analysing the nucleotide sequence content. Moreover, NGS will allow for the accurate detection of *pan-genomes* which describe the full complement of a superset of all the genes in all the strains of a species, typically applied to bacteria and archaea [14].

In the near future, sequencing techniques are expected to become even less time-consuming and more cost-effective in order to screen whole genomes within a few hours or even minutes. While sequencing techniques improve and develop overtime, the amount of data produced increases exponentially and therefore the implementation of efficient platforms to analyze and visualize such large amounts of data in fast and efficient ways has become a necessity. Following a top-down approach, the current review starts with an overview of generic visualization and analysis tools and file formats that can be used in any next generation sequencing analysis. While such tools are of a broad usage, the current review progressively focuses on their application in structural variation detection and representation and in parallel, commenting on their strengths and weaknesses, giving insights on how they could further develop to handle the overload of information and cope with the data complexity. It is not the scope of this article to describe in depth the existing sequencing techniques, but readers are strongly encouraged to follow a more detailed description about the widely used sequencing technologies in [15,16]. Thorough explanations of how hundreds of thousands or even millions of sequences can be generated by such high-throughput techniques is presented in [17,18] while sequence assembly strategies are extensively explained in [19]. The advantages and the limitations of the aforementioned techniques are discussed in [20,21].

### Sequencing technologies

#### First, second and third generation

Sequencing techniques are chronologically divided into 3 generations: the *first*, the *second* and the *third*. The key principle of the first generation (Sanger or dideoxy) sequencing techniques, which was discovered in 1977, was the use of dideoxy nucleotide triphosphates (ddNTPs) as DNA chain terminators so that the labeled fragments could be separated by size using gel electrophoresis. Dye-terminator sequencing discovered in the late 90s, utilizes labeling in a single reaction, instead of four reactions (A,T,C,G). In dye-terminator sequencing, each of the four ddNTPs is labeled with fluorescent dyes, each of which emits light at different wavelengths. Dye-terminator sequencing combined with capillary electrophoresis succeeded in speeding up performance and became one of the most standardized and widely used techniques.

Second generation high-throughput sequencing techniques generate thousands or millions of short sequences (reads) at higher speed and better accuracy. Such sequencing approaches can immediately be applied in relevant medical areas where previous Sanger-based trials fell short in capturing the desired sequencing depth in a manageable time-scale [22]. High-throughput second generation commercial technologies have already been developed by Illumina [23,24], Roche 454 [25] and Biosystems/SOLiD. Today Illumina is the most widely used platform despite its lower multiplexing capability of samples allowed [26]. Recent HiSeq Illumina systems make it possible for researchers to perform large and complex sequencing studies at a lower cost. Cutting-edge innovations can dramatically increase the number of reads, sequence output and data generation rate. Thus, researchers are now able to sequence more than five human genomes at ~30x coverage simultaneously or ~100 exome samples in a single run.

Helicos BioSciences (http://www.helicosbio.com/), Pacific Biosciences (http://www.pacificbiosciences.com/), Oxford Nanopore (http://www.nanoporetech.com/) and Complete Genomics (http://www.completegenomics.com/) belong to the third generation of sequencing techniques, each of which have their pros and cons [16,27,28]. These techniques are promising to sequence a human genome at a very low cost within a matter of hours.

While today, first generation sequencing is not used due to its forbidden cost and time consumption, second generation sequencing technologies are widely used due to their lower cost and time efficiency. Such techniques have led to a plethora of applications such as DNA-seq and assembly to determine an unknown genome from scratch or look for variations among genome samples, RNA-seq [29,30] to analyse gene expression or ChIP-seq [31] to mainly identify DNA regions that are binding sites for proteins, such as transcription factors. It is not the scope of this review to describe the aforementioned techniques into depth but we give a short description of DNA sequencing and assembly and we explain below how this can be used to discover structural variations.

### DNA sequencing and assembly

**Figure 1:**
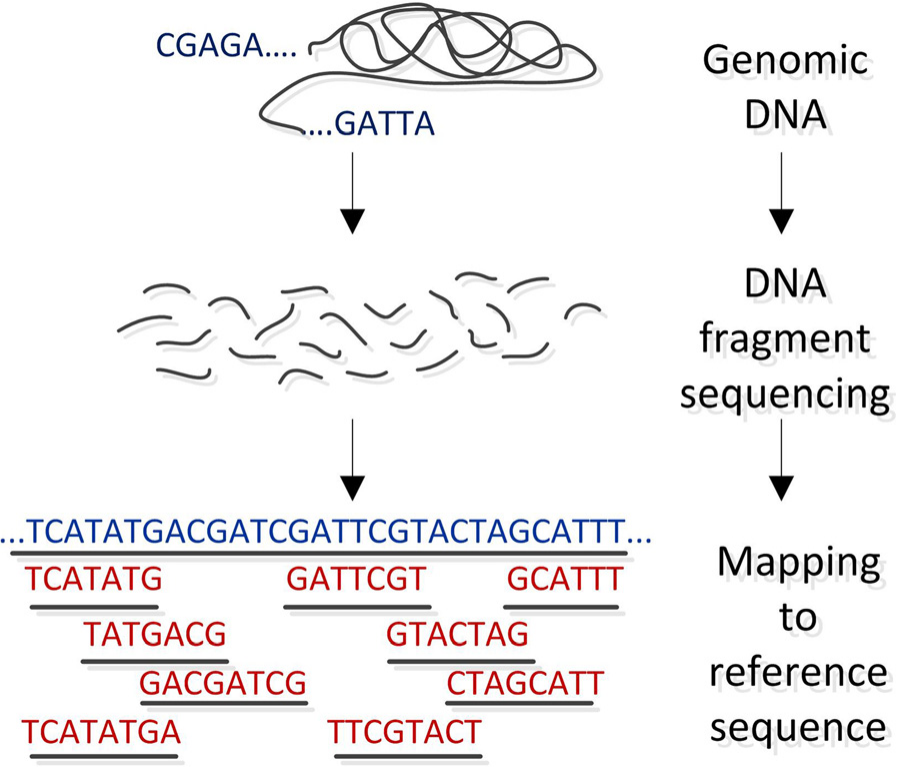
**DNA sequencing.***DNA sequencing*: 1st step: The DNA of interest is purified and extracted. 2nd step: Creation of multiple copies of DNA. 3nd step: DNA is shattered into smaller pieces. 4rd step: DNA fragment sequencing. 5th step: A computer maps the small pieces to an already known reference genome.

**Figure 2:**
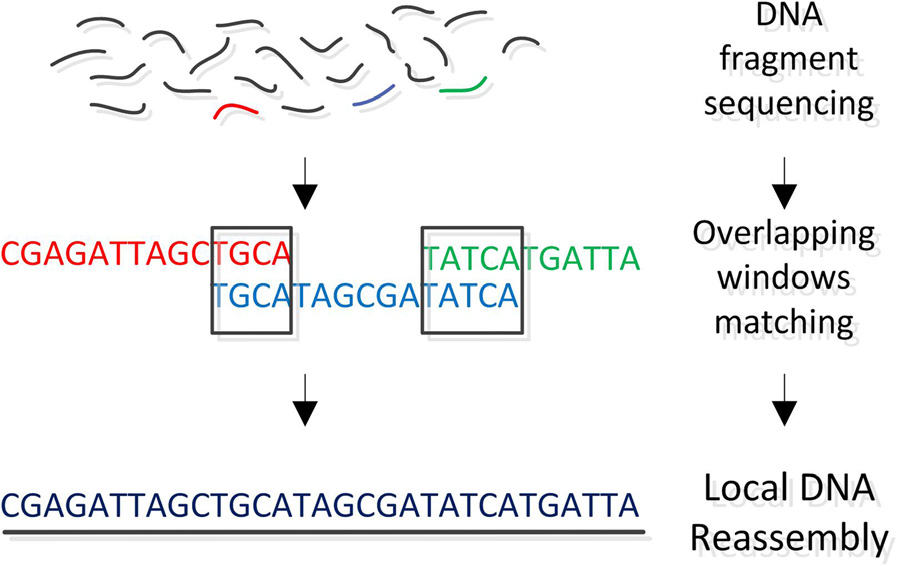
**DNA assembly.***DNA assembly*: 1st step: The DNA is purified and extracted. 2nd step: DNA is fragmented into smaller pieces. 3rd step: DNA fragment sequencing. 4th step: A computer matches the overlapping parts of the fragments to get a continuous sequence. 5th step: The whole sequence is reassembled. No prior knowledge about the DNA sequence is necessary.

DNA sequencing can be applied to very long pieces of DNA such as whole chromosomes or whole genomes, but also on targeted regions such as the exome or a selection of genes pulled-down from assays or in solution. There are two different scenarios under which DNA sequencing is carried out. In the first case a reference genome for the organism of interest already exists, whereas in the second case of *de novo* sequencing, there is no reference sequence available. The main idea behind the reference genome approach consists of 3 general steps: Firstly, DNA molecules are broken down into smaller fragments at random positions by using restriction enzymes or mechanical forces. Secondly, a sequencing library consisting of such fragments of known insert size is created, while during a third step, these fragments are sequenced and finally mapped back to an already known reference sequence. The general methodology is widely known as shotgun sequencing. The aforementioned process is depicted in Figure 1. In the case of *de novo* sequencing, where there is no a priory catalogued reference sequence for the given organism, the small sequenced fragments are assembled into *contigs* (groups of overlapping, contiguous fragments) and the consensus sequence is finally established from these contigs. This process is often compared to putting together the pieces of a jigsaw puzzle. Thus, the short DNA fragments produced are assembled electronically into one long and contiguous sequence. No prior knowledge about the original sequence is needed. While short read technologies produce higher coverage, longer reads are easier to process computationally and interpret analytically, as they are faster to align compared to short reads because they have higher significant probabilities to align to unique locations on a genome. Notable tools for sequence assembly are the: Celera [32], Atlas [33], Arachne [34], JAZZ [35], PCAP [36], ABySS [37], Velvet [38] and Phusion [39]. The accuracy of this approach increases when comparing larger sized fragments (resulting in larger overlaps) of less repetitive DNA molecules. For larger genomes, this method has many limitations mainly due to the smaller size of reads and its high cost. The aforementioned process is displayed in Figure 2.

### The structural variome

**Figure 3:**
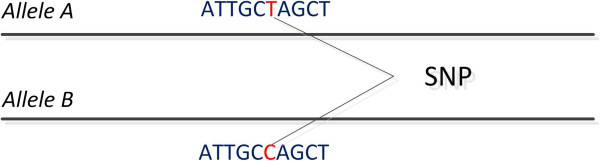
**SNP example.** A difference in a single nucleotide between two DNA fragments from different individuals. In this case we say that there are two alleles: C and T.

A *single nucleotide polymorphism* (SNP), or equally a *single nucleotide variation* (SNV), refers to a single nucleotide change (adenine-A, thymine-T, guanine-G, and cytosine-C) in genomic DNA which is observed between members of the same biological species or paired chromosomes in a single individual. A SNP example is shown in Figure 3. SNPs are single nucleotide substitutions, which are mainly divided into two types: *transitions* (interchanges of two purines or two pyrimidines such as A-G or C-T) *and transversions* (interchanges between purines and pyrimidines A-T, A-C, G-T and G-C)*.* There are multiple public databases which store information about SNPs. The National Center for Biotechnology Information (NCBI) has released dbSNP [40], a public archive for genetic variation within and across different species. The Human Gene Mutation Database (HGMD) [41] holds information about gene mutations associated with human inherited diseases and functional SNPs. The *International HapMap Project* (short for “haplotype map”) [6-10] holds information about genetic variations among people, so far from containing data from six countries. The data includes haplotypes (several SNPs that cluster together on a chromosome), their locations in the genome and their frequencies in different populations throughout the world. Other databases to be mentioned are the HGBASE [42], HGVbase [43], GWAS Central [44] and SNPedia [45]. A great variety of tools to detect SNVs and predict their impact is analytically reviewed in [46].

**Figure 4:**
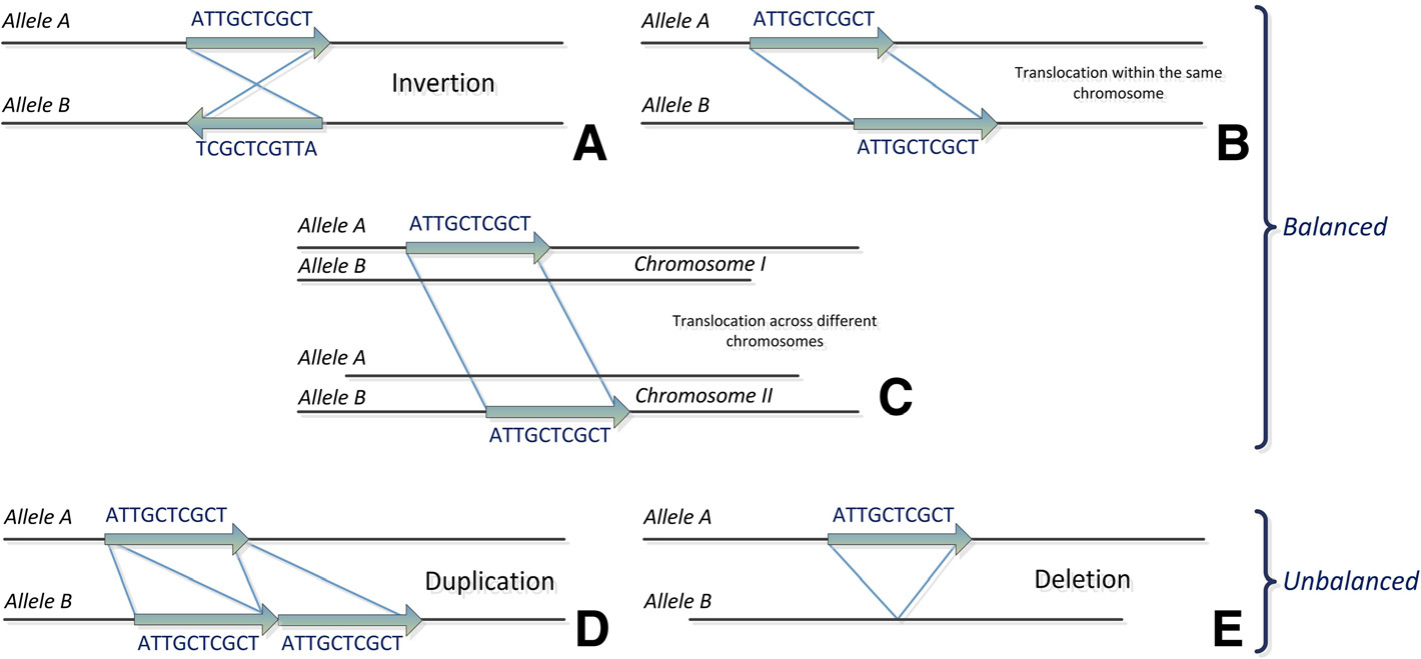
**Structural Variations.** This figure illustrates the basic structural variations. **A)** Inversion. **B)** Translocation within the same chromosome. **C)** Translocation across different chromosomes. **D)** Duplication. **E)** Deletion.

Recently, the focus has been shifted to understanding genetic differences in the form of short sequence fragments or structural rearrangements (rather than variation at the single nucleotide level). This type of variation is known as the *structural variome*. The *structural variome* refers to the set of structural genetic variations in populations of a single species that have been acquired in a relatively short time on an evolutionary scale. Structural variations are mainly separated in two categories; namely the *balanced* and the *unbalanced* variations. The basic variations include *insertions, deletions, duplications, translocations* and *inversions*. Balanced variations refer to genome rearrangements, which do not change the total content of the DNA. These are mainly inversions or intra/inter-chromosomal translocations. Unbalanced variations on the other hand, refer to rearrangements that change the total DNA content. These are insertions and deletions. Unbalanced variations are also called *copy number variations* (CNVs). Figure 4 shows a schematic representation of such intra/inter-chromosomal balanced and unbalanced structural variations.

### Methods to detect structural variations

During the past years, a great effort has been made towards the development of several techniques [47] and software applications [46] to detect structural variations in genomes. In the case of SNP detection, the differences are extracted from local alignments whereas for the detection of structural variations approaches, such as read-pair (RP), read-depth (RD) and split-reads can be used.

#### Pair-end mapping (PEM)

**Figure 5:**
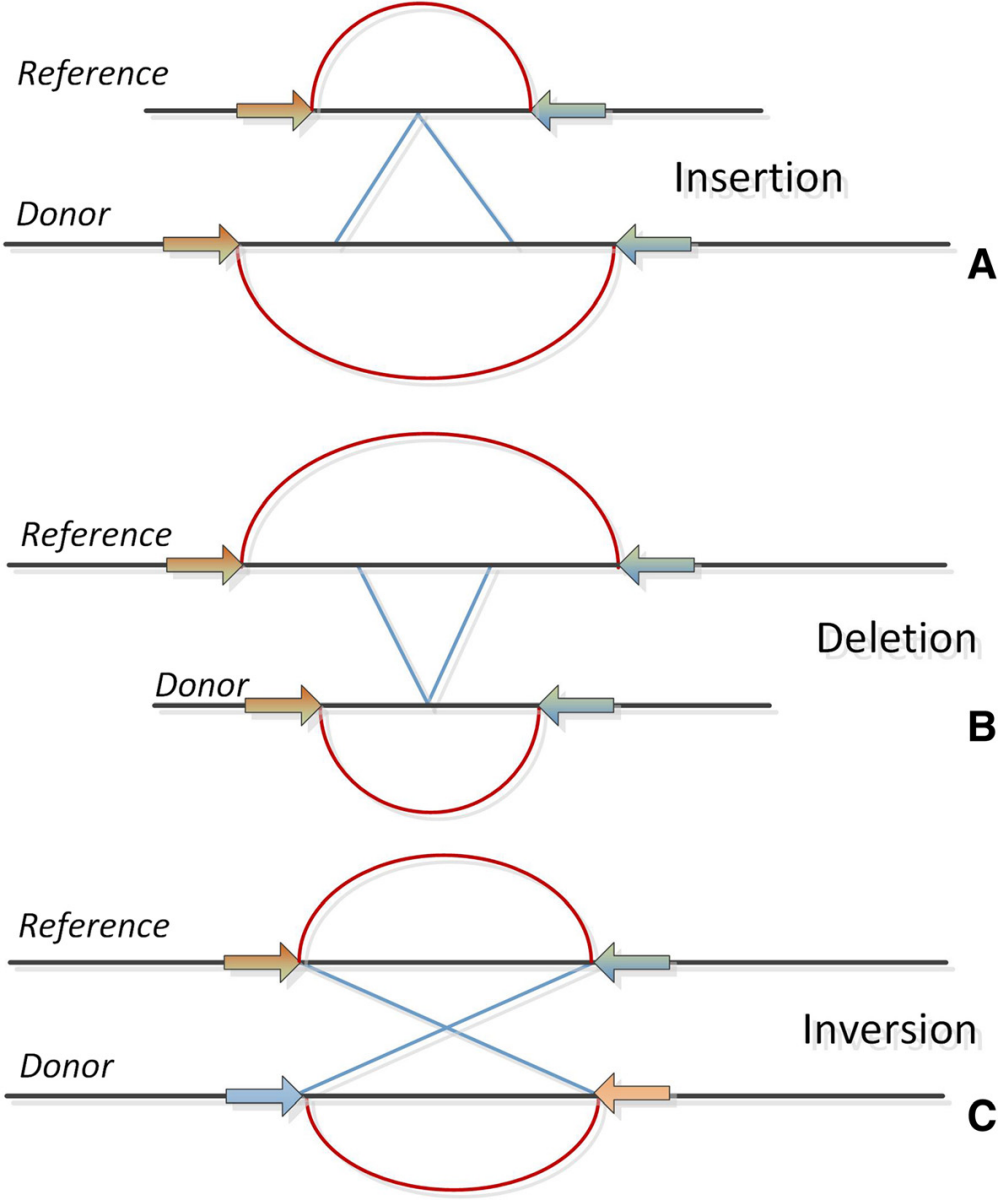
**PEM signatures.** Basic PEM signatures. **A)** Insertion. **B)** Deletion. **C)** Inversion. More PEM signatures are visually presented in [47].

According to this approach, the DNA is initially fragmented into smaller pieces. The two ends of each DNA fragment (*paired end reads* or *mate pairs*) are then sequenced and finally get mapped back to the reference sequence. Notably, the two ends of each read are long enough to allow for unique mapping back to the reference genome. The idea behind this strategy is that the ends of the reads, which align back to the reference genome, map back to specific positions of an expected distance according to information from stored DNA libraries. For certain cases, the mapping distance appears to be different from the expected length, or mapping displays an alternative orientation from that anticipated. These observations can be considered as strong indicators for the occurrence of a possible structural variation. Thus, if the mapped distance is smaller than the expected one, it could indicate a deletion or vice versa an insertion. The main difference between the terms *paired end reads* and *mate pairs*, is that while pair-end reads provide tighter insert sizes, the mate pairs give the advantage of larger insert sizes [47]. Differences and structural variations among genomes can be tracked by observing *PEM signatures*. While PEM signatures together with approaches to detect them are analytically described elsewhere [47], some common signatures are shown in Figure 5.

#### Single-end

**Figure 6:**
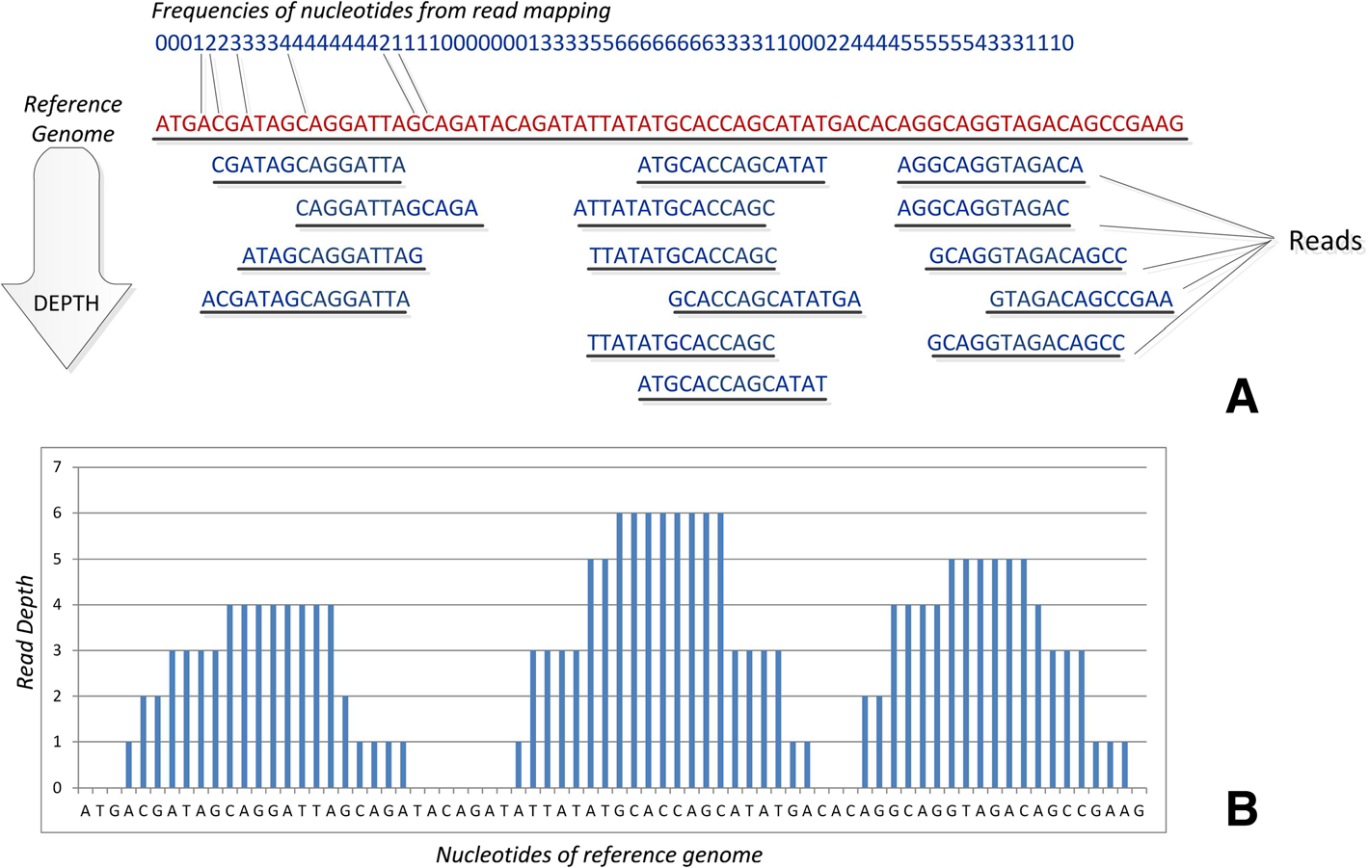
**Read depth.** Read depth: **A)** Fragments of DNA (Reads) are mapped to the original reference genome. **B)** Plotting the frequency of each nucleotide that was mapped at the reference genome.

According to this methodology, multiple copies of a DNA molecule get produced and randomly chopped into smaller fragments (*reads*). These reads are eventually aligned and mapped back to a reference genome. The reasoning behind this approach is that various reads will map back to various positions across the genome, and exhibit significant overlap of read mapping. By measuring the frequency of nucleotides mapped by the reads across the *depth of coverage* (DOC), it is possible to obtain an evaluation of the number of reads that have been mapped to a specific genomic position (see Figure 6). The *Depth of coverage* (DOC) is a significant way to detect insertions or deletions, gains or losses in a donor sample comparing to the reference genome. Thus, a region that has been deleted will have less reads mapped to it, and vice versa in cases of insertions. Similarly to PEM, the aforementioned methodology is an alternative way to extract information about possible structural variations described by DOC signatures. While read-depth has a higher resolution, it gives no information about the location of the variation and it can only detect unbalanced variations. DOC signatures, compared to PEM signatures are more suitable to detect larger events, since the stronger the event, the stronger the signal of the signature. On the other hand, PEM signatures are more suitable to detect smaller events, even with low coverage, but are far less efficient in localizing breakpoints. Available tools to detect structural variations and cluster them according to different methodologies are presented below.

#### Split-reads

According to this approach, a read is mapped to two separate locations because of possible structural variation. The prefix and the suffix of a match may be interrupted by a longer gap. This split read mapping strategy is useful for small to medium-sized rearrangements in a base pair level resolution. It is suitable for mRNA sequencing, where absent intronic arrangements can cause junction reads that span exon-exon boundaries. Often, local assembly is used to detect regions of micro-homology or non-template sequences around a breakpoint. This is done to detect the actual sequence around the break points.

### File formats

Sequencing techniques generate vast amounts of data that need to be efficiently stored, parsed and analyzed. A typical sequencing experiment might produce files ranging from few gigabytes to terabytes in size, containing thousands or millions of reads together with additional information such as read identifiers, descriptions, annotations, other meta-data, etc. Therefore, file formats such as FASTQ [48], SAM/BAM [49] or VCF [50] have been introduced to efficiently store such information.

### FASTQ

**Figure 7:**
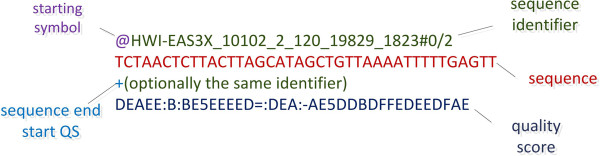
**FASTQ file.** 1st line always starts with the symbol ‘@’ followed by the sequence identifier. 2nd line contains the sequence. 3rd line starts with the symbol ‘+’ symbol which is optionally followed by the same sequence identifier and any description. It indicates the end of the sequence and the beginning of the quality score string. 4th line contains the quality score (QS) in ASCII format. The current example shows an Illumina representation.

It comes as a simple extension of the FASTA format and it is widely used in DNA sequencing mainly due to its simplicity. Its main strength is its ability to store a numeric quality score (PHRED [51]) for every nucleotide in a sequence. FASTQ mainly consists of four lines. The first line starts with the symbol ‘*@’* which is followed by the sequence identifier. The second line contains the whole sequence as a series of nucleotides in uppercase. Tabs or spaces are not permitted. The third line starts with the ‘+’ symbol which indicates the end of the sequence and the start of the quality string which follows in the 4th line. Often, the third line contains a repetition of the same identifier like in line 1. The quality string, which is shown in the 4th line, uses a subset of the ASCII printable character representation. Each character of the quality string corresponds to one nucleotide of the sequence; thus the two strings should have the same length. Encoding quality scores in ASCII format, makes FASTQ format easier to be edited. The range of printed ASCII characters to represent quality scores varies between different technologies. Sanger format accepts a PHRED quality score from 0 to 93 using ASCII 33 to 126. Illumina 1.0 encodes a Illumina quality score from −5 to 62 using ASCII 59 to 126. Illumina 1.3+ format can encode a PHRED quality score from 0 to 62 using ASCII 64 to 126. Using different ranges for every technology is often confusing, and therefore the Sanger version of the FASTQ format has found the broadest acceptance. Quality scores and how they are calculated per platform is described in [52]. A typical FASTQ file is shown in Figure 7. Compression algorithms such as [53] and [54] succeed in storing FASTQ using lower disk space. In order to interconvert files between Sanger, Illumina 1.3+ platforms, Biopython [55], EMBOSS, BioPerl [56] and BioRuby [57] come with file conversion modules.

#### Sequence alignment/Map (SAM) format

**Figure 8:**
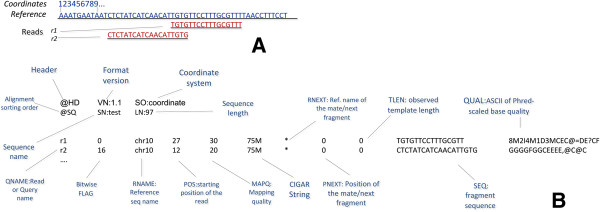
**BAM/SAM files.** Example of an alignment to the reference sequence (pileup). **A)** Read r001/1 and r001/2 constitute a read pair; r003 is a chimeric read; r004 represents a split alignment. **B)** The corresponding SAM file and their tags for each field.

It describes a flexible and a generic way to store information about alignments against a reference sequence. It supports both short and long reads produced by different sequencing platforms. It is compact in size, efficient in random access and represents the format, which was mostly used by the 1000 Genomes Project to release alignments. It mainly supports 11 mandatory and many other optional fields. For better performance, store efficiency and intensive data processing, the BAM file, a binary representation of SAM, was implemented. BAM files are compressed in the BGZF format and hold the same information as SAM, while they require less disk space. SAM can be indexed and processed by specific tools. While Figure 8 shows an example of a SAM file, a very detailed description of the SAM and BAM files is presented in [58].

#### Variant call format (VCF)

**Figure 9:**
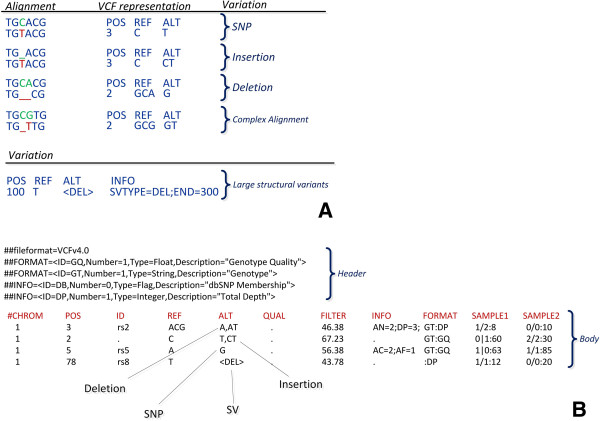
**VCF file.** This figure demonstrates an example of a CVF file. **A)** Different types of variations and polymorphisms that can be stored in CVF format. **B)** Example of a CVF format and its fields.

This specific file type was initially introduced by the 1000 Genomes Project to store the most prevalent types of sequence variation, such as SNPs and small indels (inserions/deletions) enriched by annotations. VCFtools [50] are equipped with numerous functionalities to process VCF files. Such functionalities include validations, merges and comparisons. An example of a VCF file is shown in Figure 9.

### Variant calling pipelines

Variant discovery still remains a major challenge for sequencing experiments. Bioinformatics approaches that aim to detect variations across different human genomes, have identified 3–5 million variations for each individual compared to the reference. It is noticeable that most of the current comparative sequencing-based studies are mainly targeting the exome and not the whole genome, initially due to the lower cost. It is believed that variations in the exome can have a higher chance of having a functional impact in human diseases [59]. However, recent studies show that non-coding regions contain equally important disease related information [60]. Sophisticated tools that can cope with the large data size, efficiently analyze a whole genome or an exome and accurately detect genomic variations such as deletions, insertions, inversions or inter/intra chromosomal translocation are currently necessary. Today, only few of such tools exist and are summarized in Table 1. Many of the tools are error sensitive, as false negatives in base calling may lead to the identification of non-existent variants or to missing true variants in the sample, something that still remains a bottleneck in the field.

**Table 1:** Software for predicting structural variations

**Tool**	**Single-End**	**Pair-End**	**Reference genome**	**Insertion**	**Deletion**	**Inversion**	**Translocation across chromosomes**	**Translocation within chromosome**	**Properties**	**Input File**
BreakDancer [61]		X		X	X	X	X	X	*• BreakDancerMax* for large regions and *BreakDancerMini* for indels of 10-100 bp	BAM, SAM
CNV-seq [62]	X		X	X	X				*•* Shotgun sequencing	Map locations from a BAM file (by SAM tools)
									*•* Robust statistical model	
GASV [63]		X		X	X	X	X	X	*•* Geometric approach	BAM
									*•* A SV is pictured as a polygon on a surface	
									*•* Comparison of SVs across multiple samples	
HyDRa [64]		X		X	X	X	X	X	*•* SV breakpoints by clustering discordant paired-end alignments	Tab-delimiteddiscordant paired-end mappings
MoDIL [65]		X		X	X				*•* Medium sized (10-50 bp) paired-end indels	Software specific
									*•* Able identify shorter heterozygous, as well as homozygous variants with higher accuracy	
MrFast [66]	X			X	X				*•* Short sequence reads (>25 bp)	FASTA, FASTQ
NovelSeq [67]		X		X					*•* Long novel sequence insertions	Software specific
									*•* Multiple types of variations	
PEMer [68]		X		X	X	X	X	X	*• PEMer: variations*	SVdB API
									*• SV-Simulation:* simulated paired-end reads	
									*• BreakDB: annotations*	
Pindel [69]		X	X	X	X				*•* Large deletions (1 bp–10 kb)	BAM,SAM,FASTA, FASTQ
									*•* Medium sized insertions (1–20 bp) from 36 bp paired-end short reads	
rSW-seq [70]	X		X	X	X				*•* Based on an iterative Smith-Waterman dynamic sequence alignment method	Tab-delimited file denoting the tumor/normal status for each of aligned read positions
VariationHunter [71]		X		X	X	X			*•* Evaluation of the entire possible mapping set of positions of each paired-end read and final mapping of the SVs interdependently.	Software specific
VarScan [72,73]	X		X	X	X				*•* Germline variants (SNPs and indels) in individual samples or pools of samples.	Pileup, VCF
									*•* Shared and private variants in multi-sample datasets (with mpileup).	
									*•* Somatic mutations, LOH events, and germline variants in tumor-normal pairs.	
									*•* Somatic copy number alterations (CNAs) in tumor-normal exome data.	

### Variant annotation

As genetic diseases can be caused by a variety of different possible mutations in DNA sequences, the detection of genetic variations that are associated to a specific disease of interest is very important. Even though most of the variations detected by variant callers are found to be functionally neutral [74] and do not contribute to the phenotype of a disease [75], many of them have concluded to important results. In order to better identify the causative variations for genetic disorders and characterize them, the implementation of efficient variant annotation tools emerges and is one of the most challenging aspects of the field. Table 2 summarizes the available software which serves this purpose by highlighting the strengths and the weaknesses of each application.

**Table 2:** Variant annotators

**Tool**	**Annotation**	**Data support**
Annotate-it [76]	SNPs, miRNA, Gene, Custom	OMIM, dbSNP, 200 Danish genomes, NHLBI Exomes, 1000 Genomes
KGGSeq [77]	Indels, SNPs, Gene	dbSNP, 1000 Genomes
ANNOVAR [78]	Indels, SNPs, miRNAs, Gene, Custom	dbSNP, NHLBI Exomes, 1000 Genomes
Anntools [79]	Indels, SNPs, miRNAs, Gene, Custom	dbSNP, 1000 Genomes
SeqAnt [80]	Indels, SNPs, Gene	dbSNP, 1000 Genomes
SVA [81]	Indels, SNPs, Gene, Custom	OMIM, dbSNP, 1000 Genomes
TREAT [82]	Indels, SNPs, Gene	OMIM, dbSNP, 1000 Genomes
VAAST [83]	Indels, SNPs	-
VarioWatch [84]	SNPs, Gene	OMIM, dbSNP, 1000 Genomes
Var-MD [85]	SNPs	-
VarSifter [86]	Indels, SNPs	-

### Visualization of structural variation

Visualization of high throughput data to provide meaningful views and make pattern extraction easier still remains a bottleneck in systems biology. More than ever, such applications represent a precious tool for biologists in order to allow them to directly visualize large scale data generated by sequencing. The vast amounts of data produced by deep sequencing can be impossible to analyze and visualize due to high storage, memory and screen size requirements. Therefore, the field of biological data visualization is an ever-expanding field that is required to address new tasks in order to cope with the increasing complexity of information. While a recent review [87] discusses the perspectives and the challenges of visualization approaches in sequencing, the tables below emphasize on the strengths and the weaknesses of the available tools respectively.

### Alignment tools

Aligning sequences of long length is not a trivial task. Therefore, efficient tools able to handle this load of data and provide intuitive layouts using linear or alternative representations i.e. circular are of importance. Table 3 shows a list of the widely used applications while also providing an overview of the strengths and weaknesses of each tool.

**Table 3:** Alignment tools

**Tool**	**Purpose**	**Properties**	**Support**	**Availability**
ABySS Explorer [88]	*•* Global sequence assemblies from smaller fragments of DNA	*•* de-Bruijn directed graphs	*•* DOT files [63]	*•* Java stand-alone application
CLC Genomics workbench	*•* Analysis of de novo assembly	*•* SNP detection techniques	*•* Sanger, 454, Illumina and SOLID	*•* Commercial stand-alone application
		*•* genomic rearrangements structural variations		
EagleView [89]	*•* Large genome assemblies	*•* Multiple-line scheme	*•* Navigation by genomic location, read identifiers, annotations, descriptions, user-defined coordinate map	*•* Free stand-alone application
Hawkeye [90]	*•* Detection of anomalies in data and visually identify and correct assembly errors	*•* Consensus validation of potential genes, dynamic filtering and automated clustering	*•* Compatibility with Phrap, ARACHNE [34], Celera Assembler [32] and others	*•* Free stand-alone application
LookSeq [91]	*•* Visualization of sequences derived from multiple sequencing technologies	*•* Browsing at different resolutions	*•* SAM/BAM files	*•* Web applicastion
		*•* Read-depth coverage		
		*•* Putative single nucleotide and SV		
MagicViewer [92]	*•* Assembly visualization and genetic variation annotation tool mainly developed to easily visualize short read mapping	*•* Identification and annotation of genetic variation based on the reference genome	*•* Multiple color schemes	*•* Pipeline to detect, filter, annotate visualize or classify by function genetic variations
			*•* Zoomable interface	
MapView [93]	*•* Alignments of huge-scale single-end and pair-end short reads	*•* Multiple navigation	*•* MapView formatted (MVF) files	*•* Free stand-alone application
		*•* Zooming modes		
		*•* Multi-thread processing		
		*•* Variation analysis		

### Genome browsers

Genome browsers are mainly developed to display sequencing data and genome annotations from various data sources in one common graphical interface. Initially genome browsers were mainly developed to display assemblies of smaller genomes of specific organisms, but with the latest rapid technological innovations and sequencing improvements, it is essential today to be able to navigate through sequences of huge length, and simultaneously browse for genomic annotations and other known sources of information available for these sequences. While recent studies [94-96] try to review the overlaps and comment on the future of genome browsers, we focus on the most widely used ones and we comment on their usability and their strengths as shown in Table 4.

**Table 4:** Genome browsers

**Tool**	**Purpose**	**Properties**	**Support**	**Availability**
AnnoJ [97]	*•* Deep sequencing and other genome annotation data	*•* Implemented by users, to handle data and render it into a visible form.	*•* Web 2.0 application implemented in JavaScript	*•* Web 2.0 javascript application
		*•* Plugin architecture		
		*•* Smooth navigation		
			*•* Distribution of work between the server and the client with distant access through web services	
Argo	*•* Manual annotations of complete genomes	*•* ComBo comparative viewer to view dot plots of multiple aligned sequences	*•* FASTA, Genbank, GFF, BLAST, BED, Wiggle and Genscan files	*•* Stand-alone java application that can be launched as an applet or a java web start
CGView [98]	*•* Static and interactive graphical maps of circular genomes using a circular layout	*•* Export of graphical maps in PNG, JPG or SVG formats	*•* Series of hyperlinked maps showing expanded views	Implemented in Java and it comes with its own API
			*•* XML formats	
		*•* Generation of a series of hyperlinked maps showing expanded views		
Combo [99]	*•* Dynamic browser to visualize alignments of whole genomes and their associated annotations	*•* Use of a dot plot view	*•* Zoom in and out at various resolutions	*•* Stand-alone java application
		*•* Highlighted views of detailed information from specific alignments and annotations	*•* Its own file format	
Ensembl [100,101]	*•* Annotation, analysis and display of various genomes	*•* Optimized to serve thousands users per day and handling large amounts of data	*•* API for accessing and associating genome-scale data from different species across the taxonomy	*•* Web application
GBrowse [102,103]	*•* Combination of databases and interactive web pages to manipulate and display genome annotations	*•* GBrowse_syn is an extension to show dot-plots for comparative genomics	*•* “rubber band” interface to allow faster zooming	*•* Component of the Generic Model Organism System Database Project (GMOD) [104]
				*•* HTML/Javascript
Genome Projector [105]	*•* Circular genome maps, traditional genome maps, plasmid maps, biochemical pathways maps and DNA walks	*•* Limited to bacterial species with circular chromosomes	*•* Google Maps API to offer smoother navigation and better searching functionality	*•* Web application
			*•* It comes with its own API	
IGB [106]	*•* Optimized to achieve maximum flexibility and high quality genome visualization	*•* Visualization of tiling array data, NGS results, genome annotations, microarray designs and the sequence itself	*•* Rapid navigation through multiple zooming scales and across large regions of genomic sequence	*•* Stand-alone java application
			*•* Ability to handle huge datasets and diverse data sources and formats	
IGV [107]	*•* High-performance and ability to interactively explore and integrate large datasets	*•* Sequence alignments, microarrays, and genomic annotations	*•* Great variety of input file formats	*•* Standalone application
			*•* Integration of meta-data as heatmaps for deeper analysis	
UCSC Cancer Genomics Browser [108]	*•* Integration of clinical data	*•* Heatmaps	*•* Searching capabilities to find patterns in the huge amounts of clinical and genomic data that are gathered in large-scale cancer studies	*•* Web application
		*•* Boxplots		
		*•* Proportions		
UCSC Genome Browser [109]	*•* Rapid linear visualization, examination, and querying of the data at many levels and it currently accommodates genomes of ~50 species	*• Gene Sorter*: expression, homology and other information among related groups of genes.	*•* Annotation datasets: mRNA alignments, mappings of DNA repeat elements, gene predictions, gene-expression data, disease-association data	*•* Genome Graphs for uploading and displaying genome-wide data sets
		*• Blat*: mapping any sequence to the genome while the Table Browser provides direct access to the underlying database.		
		*• VisiGene*: browsing through a large collection of in situ mouse and frog images to examine expression patterns		
			*•* Panning, zooming, and dragging capabilities increase the quality of interaction	
			*•* Uploading a large variety of files	
			*•* User specific customized sessions.	
X:map [110]	*•* Mappings between genomic features and Affymetrix microarrays	*•* Location of individual exon probes with respect to their target genes, transcripts and exons.	*•* Google Maps API to analyse and further visualize data through an associated BioConductor package	*•* Web application

### Visualization for comparative genomics

Comparative genomics is expected to be one of the main challenges of the next decade in bioinformatics research, mainly due to sequencing innovations that currently allow sequencing of whole genomes at a lower cost and a reasonable timeframe. Microbial studies, evolutionary studies and medical approaches already take advantage of such methods to compare sequences of patients against controls, newly discovered species with other closely related species and identifying the presence of specific species in a population. Therefore, a great deal of effort has been made to develop algorithms that are able to cope with multiple, pairwise and local alignments of complete genomes. Alignment of unfinished genomes, intra/inter chromosome rearrangements and identification of functional elements are some important tasks that are amenable to analysis by comparative genomics approaches. Visualization of such information is essential to obtain new knowledge and reveal patterns that can only be perceived by the human eye. In this section we present a list of lately developed software applications that aim to address all of the aforementioned tasks and we emphasize on their main functionality, their strengths and their weaknesses (see Table 5).

**Table 5:** Comparative genomics

**Tool**	**Purpose**	**Properties**	**Support**
Cinteny [111]	Fast identification of syntenic regions	*•* Flexible parameterization	*•* Pre-loaded annotated mammalian, invertebrate and fungal genomes
		*•* User-provided data such as orthologous genes, sequence tags or other markers	
ggbio [112]	Views of particular genomic regions and genome-wide overviews	*•* ideograms	*•* Bioconductor Library
		*•* grand linear views	
		*•* sequence fragment length	
		*•* edge-linked interval to data view,	
		*•* mismatch pileup,	
		*•* several splicing summaries	
GenomeComp [113]	A tool for summarizing, parsing and visualizing a genome wide sequence comparison	*•* A tool to locate the rearrangements, insertions or deletions of genome segments between species or strains	*•* Fasta format
			*•* Genbank format
			*•* EMBL format
			•BLAST output file
Circos [114]	Developed to identify and analyze similarities and differences between larger genomes	*•* Circular layout	*•* It supports its own file format
		*•* Scatter, line, and histogram plots, heat maps, tiles, connectors, and text	
DHPC [115]	Visualization of large-scale genome sequences by mapping sequences into a two-dimensional using the space-filling function of Hilbert-Peano mapping.	*•* Repeating sequences	*•* DNA sequences can be loaded in plain text or FASTA forma
		*•* Degree of base bias	
		*•* Regions of homogeneity and their boundaries,	
		*•* Mark of annotated segments such as genes or isochores.	
HilbertVis [116]	Functions to visualize long vectors of integer data by means of Hilbert curves	*•* Chip-Seq data	*•* The *stand-alone version* can load GFF, BED/Wiggle and Maq map files.
		*•* Chip-chip data	
		*•* Exploration at different zoom levels of detail	
			*•* The *R packages* HilbertVis and HilbertVisGUI are integrated in the R / Bioconductor statistical environment and can display any data vector prepared with R.
In-GAVsv [117]	Detection and visualization of structural variation from paired-end mapping data and detection of larger insertions and complex variants with lower false discovery rate	*•* Identification of different types of SVs, including large indels, inversions, translocations, tandem duplications and segmental duplications.	*•* A FASTA formatted reference sequence and a SAM alignment are required
			*•* A PTT formatted annotation file for the reference sequence is optional.
		*•* Distinction between homozygous and heterozygous variants	
Meander [118]	It is mainly developed to visually discover and explore structural variations in a genome based on Read-Depth and Pair-end information	*•* Linear view	*•* It supports its own file format both for RD and paired-end data
		*•* Hilbert curve –based view	
		*•* Comparison between up to four samples against a reference simultaneously	
		*•* Visualization ofvarious types of structural inter/intra chromosomal variations	
		*•* Exploration of data at different resolution levels	
MEDEA [119]	Genomic feature densities and genome alignments of circular genomes	*•* Customization of since tracks can by dragging and dropping into a desired position	*•* It supports its own file format
		*•* User-defined color schemes	
		*•* Zooming into specific regions and smooth navigation	
MizBee [120]	Synteny browser for exploring conservation relationships in comparative genomics data	*•* Side-by-side linked views and data visualization at different scales, from the genome to the gene	*•* Edge hustling and layering to increase visual signals about conservation relationships related to closeness, size, relationship, and orientation.
Seevolution [121]	Interactive 3D environment that enables visualization of diverse genome evolution processes	*•* Interactive animation of mutation histories involving genome rearrangement, point mutation, recombination, insertion and deletion.	*•* Accepts complete phylogenetic trees and allows path tracing between any two points.
		•Simultaneous visualization of multiple organisms related by a phylogeny.	
		•3D models of circular and linear chromosomes	
Sybil [122]	Comparative genome data, with a particular importance on protein and gene clustered data	*•* Graphical demonstration of local alignment of the genomes in which the clustered genes are located	*•* Genomes are organized in a vertical heap, as in multiple alignments and shaded areas links are used to connect genes that belong to the same cluster
VISTA [123]	Global DNA sequence alignments of arbitrary length	*•* Global and alignment visualization up to several megabases under the same scale	*•* Dynamic and interactive dot-plots

## Discussion

Advances in high throughput next generation sequencing techniques allow the production of vast amounts of data in different formats that currently cannot be analyzed in a non-automated way. Visualization approaches are today called upon to handle huge amounts of data, efficiently analyze them and deliver the knowledge to the user in a visual way that is concise, coherent and easy to comprehend and interpret. User friendliness, pattern recognition and knowledge extraction are the main goals that an optimal visualization tool should achieve. Therefore, tasks like handling the overload of information, displaying data at different resolutions, fast searching or smoother scaling and navigation are not trivial when the information to be visualized consists of millions of elements and often reaches an enormous level of complexity. Modern libraries, able to visually scale millions of data points at different resolutions are nowadays essential.

Current tools lack dynamic data structure and dynamic indexing support for better processing performance. Multi-threaded programming or parallel processing support would also be a very intuitive approach to reduce the processing time, when applications run in multicore machines with many CPUs. Efficient architecture setup, that would decentralize data and distribute the work between the servers and the clients, is also a step towards the reduction of processing time.

While knowledge is currently stored in various databases, distributed across the world and analyzed by various workflows, the need of integration among available tools is becoming a necessity. Next-generation visualization tools should be able to extract, combine and analyze knowledge and deliver it in a meaningful and sensible way. For this to happen, international standards should be defined to describe how next generation sequencing techniques should store their results and exchange them through the web. Unfortunately today, many visualization and analysis approaches are being developed independently. Many of the new methods come with their own convenient file format to store and present the information, something that will become a problem in the future when hundreds of methods will become available. Such cases are widely discussed in biological network analysis approaches [124,125].

Visual analytics in the future will play an important role to visually allow parameterizations of various workflows. So far it may be confusing and misleading to the user, when various software packages often produce significantly different results just by slightly changing the value of a single parameter. Furthermore, different approaches can come up with completely different results despite the fact that they try to answer the same question. This can be attributed to the fact that they follow a completely different methodology, therefore highlighting the need for enforcing a more general output format. Future visualization tools should offer the flexibility to easily integrate and perform fine-tuning of parameters in such a way that it allows the end users to readily adjust their research to their needs.

Finally, data integration at different levels varying from tools to concepts is a necessity. Combining functions from diverse sources varying from annotations to microarrays, RNA-Seq and ChIP-Seq data emerges towards a better understanding of the information hidden in a genome. Similarly, visual representations well established in other scientific areas, such as economics or social studies, should be shared and applied to the current field of sequencing.

## Competing interests

The authors declare that they have no competing interests.

## Authors’ contributions

The authors wrote and revised the manuscript. All authors read and approved the final manuscript.

## References

[B1] Finishing the euchromatic sequence of the human genome Nature 2004 431 7011 931 945 PMID: 15496913 10.1038/nature03001 15496913

[B2] Lander ES Linton LM Birren B Nusbaum C Zody MC Baldwin J Devon K Dewar K Doyle M FitzHugh W Initial sequencing and analysis of the human genome Nature 2001 409 6822 860 921 10.1038/35057062 11237011

[B3] Levy S Sutton G Ng PC Feuk L Halpern AL Walenz BP Axelrod N Huang J Kirkness EF Denisov G The diploid genome sequence of an individual human PLoS Biol 2007 5 10 e254 10.1371/journal.pbio.0050254 17803354 PMC1964779

[B4] Abecasis GR Altshuler D Auton A Brooks LD Durbin RM Gibbs RA Hurles ME McVean GA A map of human genome variation from population-scale sequencing Nature 2010 467 7319 1061 1073 10.1038/nature09534 20981092 PMC3042601

[B5] Abecasis GR Auton A Brooks LD DePristo MA Durbin RM Handsaker RE Kang HM Marth GT McVean GA An integrated map of genetic variation from 1,092 human genomes Nature 2012 491 7422 56 65 10.1038/nature11632 23128226 PMC3498066

[B6] Buchanan CC Torstenson ES Bush WS Ritchie MD A comparison of cataloged variation between international HapMap consortium and 1000 genomes project data J Am Med Inform Assoc 2012 19 2 289 294 10.1136/amiajnl-2011-000652 22319179 PMC3277631

[B7] Tanaka T [International HapMap project] Nihon Rinsho 2005 12 63 Suppl 29 34 16416767

[B8] Thorisson GA Smith AV Krishnan L Stein LD The international HapMap project Web site Genome Res 2005 15 11 1592 1593 10.1101/gr.4413105 16251469 PMC1310647

[B9] Integrating ethics and science in the international HapMap project Nat Rev Genet 2004 5 6 467 475 PMID: 15153999 10.1038/nrg1351 15153999 PMC2271136

[B10] The international HapMap project Nature 2003 426 6968 789 796 PMID: 14685227 10.1038/nature02168 14685227

[B11] Pitman NC Jorgensen PM Estimating the size of the world's threatened flora Science 2002 298 5595 989 10.1126/science.298.5595.989 12411696

[B12] Weigel D Mott R The 1001 genomes project for arabidopsis thaliana Genome Biol 2009 10 5 107 10.1186/gb-2009-10-5-107 19519932 PMC2718507

[B13] Genome 10K: a proposal to obtain whole-genome sequence for 10,000 vertebrate species J Hered 2009 100 6 659 674 PMID: 19892720 19892720 10.1093/jhered/esp086 PMC2877544

[B14] Medini D Donati C Tettelin H Masignani V Rappuoli R The microbial pan-genome Curr Opin Genet Dev 2005 15 6 589 594 10.1016/j.gde.2005.09.006 16185861

[B15] Cullum R Alder O Hoodless PA The next generation: using new sequencing technologies to analyse gene regulation Respirology 2011 16 2 210 222 10.1111/j.1440-1843.2010.01899.x 21077988

[B16] Metzker ML Sequencing technologies - the next generation Nat Rev Genet 2010 11 1 31 46 10.1038/nrg2626 19997069

[B17] Church M Genomes for All Sci Am 2006 294 46 54 16468433 10.1038/scientificamerican0106-46

[B18] Hall N Advanced sequencing technologies and their wider impact in microbiology J Exp Biol 2007 210 Pt 9 1518 1525 17449817 10.1242/jeb.001370

[B19] Nagarajan N Pop M Sequencing and genome assembly using next-generation technologies Methods Mol Biol 2010 673 1 17 10.1007/978-1-60761-842-3_1 20835789

[B20] Git A Dvinge H Salmon-Divon M Osborne M Kutter C Hadfield J Bertone P Caldas C Systematic comparison of microarray profiling, real-time PCR, and next-generation sequencing technologies for measuring differential microRNA expression RNA 2010 16 5 991 1006 10.1261/rna.1947110 20360395 PMC2856892

[B21] Hert DG Fredlake CP Barron AE Advantages and limitations of next-generation sequencing technologies: a comparison of electrophoresis and non-electrophoresis methods Electrophoresis 2008 29 23 4618 4626 10.1002/elps.200800456 19053153

[B22] Thomas RK Baker AC Debiasi RM Winckler W Laframboise T Lin WM Wang M Feng W Zander T MacConaill L High-throughput oncogene mutation profiling in human cancer Nat Genet 2007 39 3 347 351 10.1038/ng1975 17293865

[B23] Bennett S Solexa Ltd Pharmacogenomics 2004 5 4 433 438 10.1517/14622416.5.4.433 15165179

[B24] Bentley DR Balasubramanian S Swerdlow HP Smith GP Milton J Brown CG Hall KP Evers DJ Barnes CL Bignell HR Accurate whole human genome sequencing using reversible terminator chemistry Nature 2008 456 7218 53 59 10.1038/nature07517 18987734 PMC2581791

[B25] Margulies M Egholm M Altman WE Attiya S Bader JS Bemben LA Berka J Braverman MS Chen YJ Chen Z Genome sequencing in microfabricated high-density picolitre reactors Nature 2005 437 7057 376 380 16056220 10.1038/nature03959 PMC1464427

[B26] Luo C Tsementzi D Kyrpides N Read T Konstantinidis KT Direct comparisons of illumina vs. Roche 454 sequencing technologies on the same microbial community DNA sample PLoS One 2012 7 2 e30087 10.1371/journal.pone.0030087 22347999 PMC3277595

[B27] Liu L Li Y Li S Hu N He Y Pong R Lin D Lu L Law M Comparison of next-generation sequencing systems J Biomed Biotechnol 2012 2012 251364 22829749 10.1155/2012/251364 PMC3398667

[B28] Xu M Fujita D Hanagata N Perspectives and challenges of emerging single-molecule DNA sequencing technologies Small 2009 5 23 2638 2649 10.1002/smll.200900976 19904762

[B29] Wang Z Gerstein M Snyder M RNA-Seq: a revolutionary tool for transcriptomics Nat Rev Genet 2009 10 1 57 63 10.1038/nrg2484 19015660 PMC2949280

[B30] Morin R Bainbridge M Fejes A Hirst M Krzywinski M Pugh T McDonald H Varhol R Jones S Marra M Profiling the HeLa S3 transcriptome using randomly primed cDNA and massively parallel short-read sequencing Biotechniques 2008 45 1 81 94 10.2144/000112900 18611170

[B31] Furey TS ChIP-seq and beyond: new and improved methodologies to detect and characterize protein-DNA interactions Nat Rev Genet 2012 13 12 840 852 10.1038/nrg3306 23090257 PMC3591838

[B32] Myers EW Sutton GG Delcher AL Dew IM Fasulo DP Flanigan MJ Kravitz SA Mobarry CM Reinert KH Remington KA A whole-genome assembly of drosophila Science 2000 287 5461 2196 2204 10.1126/science.287.5461.2196 10731133

[B33] Havlak P Chen R Durbin KJ Egan A Ren Y Song XZ Weinstock GM Gibbs RA The atlas genome assembly system Genome Res 2004 14 4 721 732 10.1101/gr.2264004 15060016 PMC383319

[B34] Batzoglou S Jaffe DB Stanley K Butler J Gnerre S Mauceli E Berger B Mesirov JP Lander ES ARACHNE: a whole-genome shotgun assembler Genome Res 2002 12 1 177 189 10.1101/gr.208902 11779843 PMC155255

[B35] Aparicio S Chapman J Stupka E Putnam N Chia JM Dehal P Christoffels A Rash S Hoon S Smit A Whole-genome shotgun assembly and analysis of the genome of fugu rubripes Science 2002 297 5585 1301 1310 10.1126/science.1072104 12142439

[B36] Huang X Wang J Aluru S Yang SP Hillier L PCAP: a whole-genome assembly program Genome Res 2003 13 9 2164 2170 10.1101/gr.1390403 12952883 PMC403719

[B37] Simpson JT Wong K Jackman SD Schein JE Jones SJ Birol I ABySS: a parallel assembler for short read sequence data Genome Res 2009 19 6 1117 1123 10.1101/gr.089532.108 19251739 PMC2694472

[B38] Zerbino DR Birney E Velvet: algorithms for de novo short read assembly using de bruijn graphs Genome Res 2008 18 5 821 829 10.1101/gr.074492.107 18349386 PMC2336801

[B39] Mullikin JC Ning Z The phusion assembler Genome Res 2003 13 1 81 90 10.1101/gr.731003 12529309 PMC430959

[B40] Wheeler DL Barrett T Benson DA Bryant SH Canese K Chetvernin V Church DM DiCuccio M Edgar R Federhen S Database resources of the national center for biotechnology information Nucleic Acids Res 2007 35 Database issue D5 12 17170002 10.1093/nar/gkl1031 PMC1781113

[B41] Stenson PD Ball EV Mort M Phillips AD Shaw K Cooper DN Baxevanis AD The human gene mutation database (HGMD) and its exploitation in the fields of personalized genomics and molecular evolution Current protocols in bioinformatics 2012 Chapter 1:Unit1 13. PMID:22948725 10.1002/0471250953.bi0113s39 22948725

[B42] Brookes AJ Lehvaslaiho H Siegfried M Boehm JG Yuan YP Sarkar CM Bork P Ortigao F HGBASE: a database of SNPs and other variations in and around human genes Nucleic Acids Res 2000 28 1 356 360 10.1093/nar/28.1.356 10592273 PMC102467

[B43] Fredman D Siegfried M Yuan YP Bork P Lehvaslaiho H Brookes AJ HGVbase: a human sequence variation database emphasizing data quality and a broad spectrum of data sources Nucleic Acids Res 2002 30 1 387 391 10.1093/nar/30.1.387 11752345 PMC99093

[B44] The GWAS centralhttp://www.gwascentral.org

[B45] The SNPedia http://www.snpedia.com/index.php/SNPedia

[B46] Karchin R Next generation tools for the annotation of human SNPs Brief Bioinform 2009 10 1 35 52 19181721 10.1093/bib/bbn047 PMC2638621

[B47] Medvedev P Stanciu M Brudno M Computational methods for discovering structural variation with next-generation sequencing Nat Methods 2009 6 11 Suppl S13 20 19844226 10.1038/nmeth.1374

[B48] Cock PJ Fields CJ Goto N Heuer ML Rice PM The sanger FASTQ file format for sequences with quality scores, and the solexa/illumina FASTQ variants Nucleic Acids Res 2010 38 6 1767 1771 10.1093/nar/gkp1137 20015970 PMC2847217

[B49] Li H Handsaker B Wysoker A Fennell T Ruan J Homer N Marth G Abecasis G Durbin R Genome project data processing S: the sequence alignment/Map format and SAMtools Bioinformatics 2009 25 16 2078 2079 10.1093/bioinformatics/btp352 19505943 PMC2723002

[B50] Danecek P Auton A Abecasis G Albers CA Banks E DePristo MA Handsaker RE Lunter G Marth GT Sherry ST The variant call format and VCFtools Bioinformatics 2011 27 15 2156 2158 10.1093/bioinformatics/btr330 21653522 PMC3137218

[B51] Ewing B Hillier L Wendl MC Green P Base-calling of automated sequencer traces using phred. I. Accuracy assessment Genome Res 1998 8 3 175 185 10.1101/gr.8.3.175 9521921

[B52] Pleasance ED Cheetham RK Stephens PJ McBride DJ Humphray SJ Greenman CD Varela I Lin ML Ordonez GR Bignell GR A comprehensive catalogue of somatic mutations from a human cancer genome Nature 2010 463 7278 191 196 10.1038/nature08658 20016485 PMC3145108

[B53] Deorowicz S Grabowski S Compression of genomic sequences in FASTQ format Bioinformatics 2011 PMID: 21252073 10.1093/bioinformatics/btr014 21252073

[B54] Tembe W Lowey J Suh E G-SQZ: compact encoding of genomic sequence and quality data Bioinformatics 2010 26 17 2192 2194 10.1093/bioinformatics/btq346 20605925

[B55] Cock PJ Antao T Chang JT Chapman BA Cox CJ Dalke A Friedberg I Hamelryck T Kauff F Wilczynski B Biopython: freely available python tools for computational molecular biology and bioinformatics Bioinformatics 2009 25 11 1422 1423 10.1093/bioinformatics/btp163 19304878 PMC2682512

[B56] Stajich JE Block D Boulez K Brenner SE Chervitz SA Dagdigian C Fuellen G Gilbert JG Korf I Lapp H The bioperl toolkit: perl modules for the life sciences Genome Res 2002 12 10 1611 1618 10.1101/gr.361602 12368254 PMC187536

[B57] Goto N Prins P Nakao M Bonnal R Aerts J Katayama T BioRuby: bioinformatics software for the ruby programming language Bioinformatics 2010 26 20 2617 2619 10.1093/bioinformatics/btq475 20739307 PMC2951089

[B58] Li H Handsaker B Wysoker A Fennell T Ruan J Homer N Marth G Abecasis G Durbin R The sequence alignment/Map format and SAMtools Bioinformatics 2009 25 16 2078 2079 10.1093/bioinformatics/btp352 19505943 PMC2723002

[B59] Botstein D Risch N Discovering genotypes underlying human phenotypes: past successes for mendelian disease, future approaches for complex disease Nat Genet 2003 33 Suppl 228 237 12610532 10.1038/ng1090

[B60] Altshuler D Daly MJ Lander ES Genetic mapping in human disease Science 2008 322 5903 881 888 10.1126/science.1156409 18988837 PMC2694957

[B61] Chen K Wallis JW McLellan MD Larson DE Kalicki JM Pohl CS McGrath SD Wendl MC Zhang Q Locke DP BreakDancer: an algorithm for high-resolution mapping of genomic structural variation Nat Methods 2009 6 9 677 681 10.1038/nmeth.1363 19668202 PMC3661775

[B62] Xie C Tammi MT CNV-seq, a new method to detect copy number variation using high-throughput sequencing BMC Bioinforma 2009 10 80 10.1186/1471-2105-10-80 PMC2667514 19267900

[B63] Sindi S Helman E Bashir A Raphael BJ A geometric approach for classification and comparison of structural variants Bioinformatics 2009 25 12 i222 230 10.1093/bioinformatics/btp208 19477992 PMC2687962

[B64] Quinlan AR Clark RA Sokolova S Leibowitz ML Zhang Y Hurles ME Mell JC Hall IM Genome-wide mapping and assembly of structural variant breakpoints in the mouse genome Genome Res 2010 20 5 623 635 10.1101/gr.102970.109 20308636 PMC2860164

[B65] Lee S Hormozdiari F Alkan C Brudno M MoDIL: detecting small indels from clone-end sequencing with mixtures of distributions Nat Methods 2009 6 7 473 474 10.1038/nmeth.f.256 19483690

[B66] Hach F Hormozdiari F Alkan C Hormozdiari F Birol I Eichler EE Sahinalp SC mrsFAST: a cache-oblivious algorithm for short-read mapping Nat Methods 2010 7 8 576 577 10.1038/nmeth0810-576 20676076 PMC3115707

[B67] Hajirasouliha I Hormozdiari F Alkan C Kidd JM Birol I Eichler EE Sahinalp SC Detection and characterization of novel sequence insertions using paired-end next-generation sequencing Bioinformatics 2010 26 10 1277 1283 10.1093/bioinformatics/btq152 20385726 PMC2865866

[B68] Korbel JO Abyzov A Mu XJ Carriero N Cayting P Zhang Z Snyder M Gerstein MB PEMer: a computational framework with simulation-based error models for inferring genomic structural variants from massive paired-end sequencing data Genome Biol 2009 10 2 R23 10.1186/gb-2009-10-2-r23 19236709 PMC2688268

[B69] Ye K Schulz MH Long Q Apweiler R Ning Z Pindel: a pattern growth approach to detect break points of large deletions and medium sized insertions from paired-end short reads Bioinformatics 2009 25 21 2865 2871 10.1093/bioinformatics/btp394 19561018 PMC2781750

[B70] Kim TM Luquette LJ Xi R Park PJ rSW-seq: algorithm for detection of copy number alterations in deep sequencing data BMC Bioinforma 2010 11 432 10.1186/1471-2105-11-432 PMC2939611 20718989

[B71] Hormozdiari F Hajirasouliha I Dao P Hach F Yorukoglu D Alkan C Eichler EE Sahinalp SC Next-generation VariationHunter: combinatorial algorithms for transposon insertion discovery Bioinformatics 2010 26 12 i350 357 10.1093/bioinformatics/btq216 20529927 PMC2881400

[B72] Koboldt DC Zhang Q Larson DE Shen D McLellan MD Lin L Miller CA Mardis ER Ding L Wilson RK VarScan 2: somatic mutation and copy number alteration discovery in cancer by exome sequencing Genome Res 2012 22 3 568 576 10.1101/gr.129684.111 22300766 PMC3290792

[B73] Koboldt DC Chen K Wylie T Larson DE McLellan MD Mardis ER Weinstock GM Wilson RK Ding L VarScan: variant detection in massively parallel sequencing of individual and pooled samples Bioinformatics 2009 25 17 2283 2285 10.1093/bioinformatics/btp373 19542151 PMC2734323

[B74] McClellan J King MC Genetic heterogeneity in human disease Cell 2010 141 2 210 217 10.1016/j.cell.2010.03.032 20403315

[B75] Cantor RM Lange K Sinsheimer JS Prioritizing GWAS results: a review of statistical methods and recommendations for their application Am J Hum Genet 2010 86 1 6 22 10.1016/j.ajhg.2009.11.017 20074509 PMC2801749

[B76] Sifrim A Van Houdt JK Tranchevent LC Nowakowska B Sakai R Pavlopoulos GA Devriendt K Vermeesch JR Moreau Y Aerts J Annotate-it: a swiss-knife approach to annotation, analysis and interpretation of single nucleotide variation in human disease Genome Med 2012 4 9 73 10.1186/gm374 23013645 PMC3580443

[B77] Li MX Gui HS Kwan JS Bao SY Sham PC A comprehensive framework for prioritizing variants in exome sequencing studies of mendelian diseases Nucleic Acids Res 2012 40 7 e53 10.1093/nar/gkr1257 22241780 PMC3326332

[B78] Wang K Li M Hakonarson H ANNOVAR: functional annotation of genetic variants from high-throughput sequencing data Nucleic Acids Res 2010 38 16 e164 10.1093/nar/gkq603 20601685 PMC2938201

[B79] Makarov V O'Grady T Cai G Lihm J Buxbaum JD Yoon S AnnTools: a comprehensive and versatile annotation toolkit for genomic variants Bioinformatics 2012 28 5 724 725 10.1093/bioinformatics/bts032 22257670 PMC3289923

[B80] Shetty AC Athri P Mondal K Horner VL Steinberg KM Patel V Caspary T Cutler DJ Zwick ME SeqAnt: a web service to rapidly identify and annotate DNA sequence variations BMC Bioinforma 2010 11 471 10.1186/1471-2105-11-471 PMC2955049 20854673

[B81] Ge D Ruzzo EK Shianna KV He M Pelak K Heinzen EL Need AC Cirulli ET Maia JM Dickson SP SVA: software for annotating and visualizing sequenced human genomes Bioinformatics 2011 27 14 1998 2000 10.1093/bioinformatics/btr317 21624899 PMC3129530

[B82] Asmann YW Middha S Hossain A Baheti S Li Y Chai HS Sun Z Duffy PH Hadad AA Nair A TREAT: a bioinformatics tool for variant annotations and visualizations in targeted and exome sequencing data Bioinformatics 2012 28 2 277 278 10.1093/bioinformatics/btr612 22088845 PMC3259432

[B83] Yandell M Huff C Hu H Singleton M Moore B Xing J Jorde LB Reese MG A probabilistic disease-gene finder for personal genomes Genome Res 2011 21 9 1529 1542 10.1101/gr.123158.111 21700766 PMC3166837

[B84] Cheng YC Hsiao FC Yeh EC Lin WJ Tang CY Tseng HC Wu HT Liu CK Chen CC Chen YT VarioWatch: providing large-scale and comprehensive annotations on human genomic variants in the next generation sequencing era Nucleic Acids Res 2012 40 Web Server issue W76 81 22618869 10.1093/nar/gks397 PMC3394242

[B85] Sincan M Simeonov DR Adams D Markello TC Pierson TM Toro C Gahl WA Boerkoel CF VAR-MD: a tool to analyze whole exome-genome variants in small human pedigrees with mendelian inheritance Hum Mutat 2012 33 4 593 598 10.1002/humu.22034 22290570

[B86] Teer JK Green ED Mullikin JC Biesecker LG VarSifter: visualizing and analyzing exome-scale sequence variation data on a desktop computer Bioinformatics 2012 28 4 599 600 10.1093/bioinformatics/btr711 22210868 PMC3278764

[B87] O'Donoghue SI Gavin AC Gehlenborg N Goodsell DS Heriche JK Nielsen CB North C Olson AJ Procter JB Shattuck DW Visualizing biological data-now and in the future Nat Methods 2010 7 3 Suppl S2 4 20195254 10.1038/nmeth.f.301

[B88] Nielsen CB Jackman SD Birol I Jones SJ ABySS-explorer: visualizing genome sequence assemblies IEEE Trans Vis Comput Graph 2009 15 6 881 888 19834150 10.1109/TVCG.2009.116

[B89] Huang W Marth G EagleView: a genome assembly viewer for next-generation sequencing technologies Genome Res 2008 18 9 1538 1543 10.1101/gr.076067.108 18550804 PMC2527701

[B90] Schatz MC Phillippy AM Sommer DD Delcher AL Puiu D Narzisi G Salzberg SL Pop M Hawkeye and AMOS: visualizing and assessing the quality of genome assemblies Brief Bioinform 2013 14 2 213 224 10.1093/bib/bbr074 22199379 PMC3603210

[B91] Manske HM Kwiatkowski DP LookSeq: a browser-based viewer for deep sequencing data Genome Res 2009 19 11 2125 2132 10.1101/gr.093443.109 19679872 PMC2775587

[B92] Hou H Zhao F Zhou L Zhu E Teng H Li X Bao Q Wu J Sun Z MagicViewer: integrated solution for next-generation sequencing data visualization and genetic variation detection and annotation Nucleic Acids Res 2010 38 Web Server issue W732 736 20444865 10.1093/nar/gkq302 PMC2896176

[B93] Bao H Guo H Wang J Zhou R Lu X Shi S MapView: visualization of short reads alignment on a desktop computer Bioinformatics 2009 25 12 1554 1555 10.1093/bioinformatics/btp255 19369497

[B94] Furey TS Comparison of human (and other) genome browsers Hum Genomics 2006 2 4 266 270 10.1186/1479-7364-2-4-266 16460652 PMC3525149

[B95] Cline MS Kent WJ Understanding genome browsing Nat Biotechnol 2009 27 2 153 155 10.1038/nbt0209-153 19204697

[B96] Nielsen CB Cantor M Dubchak I Gordon D Wang T Visualizing genomes: techniques and challenges Nat Methods 2010 7 3 Suppl S5 S15 20195257 10.1038/nmeth.1422

[B97] AnnoJhttp://www.annoj.org

[B98] Grant JR Stothard P The CGView server: a comparative genomics tool for circular genomes Nucleic Acids Res 2008 36 Web Server issue W181 184 18411202 10.1093/nar/gkn179 PMC2447734

[B99] Engels R Yu T Burge C Mesirov JP DeCaprio D Galagan JE Combo: a whole genome comparative browser Bioinformatics 2006 22 14 1782 1783 10.1093/bioinformatics/btl193 16709588

[B100] Flicek P Amode MR Barrell D Beal K Brent S Carvalho-Silva D Clapham P Coates G Fairley S Fitzgerald S Ensembl 2012 Nucleic Acids Res 2012 40 Database issue D84 90 22086963 10.1093/nar/gkr991 PMC3245178

[B101] Hubbard T Barker D Birney E Cameron G Chen Y Clark L Cox T Cuff J Curwen V Down T The ensembl genome database project Nucleic Acids Res 2002 30 1 38 41 10.1093/nar/30.1.38 11752248 PMC99161

[B102] Papanicolaou A Heckel DG The GMOD drupal bioinformatic server framework Bioinformatics 2010 26 24 3119 3124 10.1093/bioinformatics/btq599 20971988 PMC2995126

[B103] Wang H Su Y Mackey AJ Kraemer ET Kissinger JC SynView: a GBrowse-compatible approach to visualizing comparative genome data Bioinformatics 2006 22 18 2308 2309 10.1093/bioinformatics/btl389 16844709

[B104] Stein LD Mungall C Shu S Caudy M Mangone M Day A Nickerson E Stajich JE Harris TW Arva A The generic genome browser: a building block for a model organism system database Genome Res 2002 12 10 1599 1610 10.1101/gr.403602 12368253 PMC187535

[B105] Arakawa K Tamaki S Kono N Kido N Ikegami K Ogawa R Tomita M Genome projector: zoomable genome map with multiple views BMC Bioinforma 2009 10 31 10.1186/1471-2105-10-31 PMC2636772 19166610

[B106] Nicol JW Helt GA Blanchard SG Jr Raja A Loraine AE The integrated genome browser: free software for distribution and exploration of genome-scale datasets Bioinformatics 2009 25 20 2730 2731 10.1093/bioinformatics/btp472 19654113 PMC2759552

[B107] Thorvaldsdottir H Robinson JT Mesirov JP Integrative genomics viewer (IGV): high-performance genomics data visualization and exploration Brief Bioinform 2013 14 2 178 192 10.1093/bib/bbs017 22517427 PMC3603213

[B108] Zhu J Sanborn JZ Benz S Szeto C Hsu F Kuhn RM Karolchik D Archie J Lenburg ME Esserman LJ The UCSC cancer genomics browser Nat Methods 2009 6 4 239 240 10.1038/nmeth0409-239 19333237 PMC5027375

[B109] Kent WJ Sugnet CW Furey TS Roskin KM Pringle TH Zahler AM Haussler D The human genome browser at UCSC Genome Res 2002 12 6 996 1006 12045153 10.1101/gr.229102 PMC186604

[B110] Yates T Okoniewski MJ Miller CJ X:Map: annotation and visualization of genome structure for affymetrix exon array analysis Nucleic Acids Res 2008 36 Database issue D780 786 17932061 10.1093/nar/gkm779 PMC2238884

[B111] Sinha AU Meller J Cinteny: flexible analysis and visualization of synteny and genome rearrangements in multiple organisms BMC Bioinforma 2007 8 82 10.1186/1471-2105-8-82 PMC1821339 17343765

[B112] Yin T Cook D Lawrence M Ggbio: an R package for extending the grammar of graphics for genomic data Genome Biol 2012 13 8 R77 10.1186/gb-2012-13-8-r77 22937822 PMC4053745

[B113] Yang J Wang J Yao ZJ Jin Q Shen Y Chen R GenomeComp: a visualization tool for microbial genome comparison J Microbiol Methods 2003 54 3 423 426 10.1016/S0167-7012(03)00094-0 12842490

[B114] Krzywinski M Schein J Birol I Connors J Gascoyne R Horsman D Jones SJ Marra MA Circos: an information aesthetic for comparative genomics Genome Res 2009 19 9 1639 1645 10.1101/gr.092759.109 19541911 PMC2752132

[B115] Deng X Rayner S Liu X Zhang Q Yang Y Li N DHPC: a new tool to express genome structural features Genomics 2008 91 5 476 483 10.1016/j.ygeno.2008.01.003 18343093

[B116] Anders S Visualization of genomic data with the hilbert curve Bioinformatics 2009 25 10 1231 1235 PMID: 23605045 10.1093/bioinformatics/btp152 19297348 PMC2677744

[B117] Qi J Zhao F InGAP-sv: a novel scheme to identify and visualize structural variation from paired end mapping data Nucleic Acids Res 2011 39 Web Server issue W567 575 21715388 10.1093/nar/gkr506 PMC3125812

[B118] Pavlopoulos GA Kumar P Sifrim A Sakai R Lin ML Voet T Moreau Y Aerts J Meander: visually exploring the structural variome using space-filling curves Nucleic Acids Res 2013 10.1093/nar/gkt254 PMC3675473 23605045

[B119] MEDEA Comparative genomic visualization with adobe flash[http://www.broadinstitute.org/annotation/medea/]

[B120] Meyer M Munzner T Pfister H MizBee: a multiscale synteny browser IEEE Trans Vis Comput Graph 2009 15 6 897 904 19834152 10.1109/TVCG.2009.167

[B121] Esteban-Marcos A Darling AE Ragan MA Seevolution: visualizing chromosome evolution Bioinformatics 2009 25 7 960 961 10.1093/bioinformatics/btp096 19233896 PMC2660879

[B122] Crabtree J Angiuoli SV Wortman JR White OR Sybil: methods and software for multiple genome comparison and visualization Methods Mol Biol 2007 408 93 108 Clifton, NJ 10.1007/978-1-59745-547-3_6 18314579

[B123] Mayor C Brudno M Schwartz JR Poliakov A Rubin EM Frazer KA Pachter LS Dubchak I VISTA : visualizing global DNA sequence alignments of arbitrary length Bioinformatics 2000 16 11 1046 1047 10.1093/bioinformatics/16.11.1046 11159318

[B124] Pavlopoulos GA Soldatos TG Barbosa-Silva A Schneider R A reference guide for tree analysis and visualization BioData Min 2010 3 1 1 10.1186/1756-0381-3-1 20175922 PMC2844399

[B125] Pavlopoulos GA Wegener AL Schneider R A survey of visualization tools for biological network analysis BioData Min 2008 1 12 10.1186/1756-0381-1-12 19040716 PMC2636684

